# A Novel Feature Selection Method Based on Extreme Learning Machine and Fractional-Order Darwinian PSO

**DOI:** 10.1155/2018/5078268

**Published:** 2018-05-06

**Authors:** Yuan-Yuan Wang, Huan Zhang, Chen-Hui Qiu, Shun-Ren Xia

**Affiliations:** ^1^Key Laboratory of Biomedical Engineering of Ministry of Education, Zhejiang University, Hangzhou, China; ^2^Zhejiang Provincial Key Laboratory of Cardio-Cerebral Vascular Detection Technology and Medicinal Effectiveness Appraisal, Hangzhou, China

## Abstract

The paper presents a novel approach for feature selection based on extreme learning machine (ELM) and Fractional-order Darwinian particle swarm optimization (FODPSO) for regression problems. The proposed method constructs a fitness function by calculating mean square error (MSE) acquired from ELM. And the optimal solution of the fitness function is searched by an improved particle swarm optimization, FODPSO. In order to evaluate the performance of the proposed method, comparative experiments with other relative methods are conducted in seven public datasets. The proposed method obtains six lowest MSE values among all the comparative methods. Experimental results demonstrate that the proposed method has the superiority of getting lower MSE with the same scale of feature subset or requiring smaller scale of feature subset for similar MSE.

## 1. Introduction

In the field of artificial intelligence, more and more variables or features are involved. An excessive set of features may lead to lower computation accuracy, slower speed, and additional memory occupation. Feature selection is used to choose smaller but sufficient feature subsets, to improve or at least not significantly harm the predicting accuracy in the meantime. Many studies have been conducted to optimize feature selections [1–4]. As far as we know, there are two key points in search-based feature selection process: learning algorithms and optimization algorithms. Many techniques could be involved in this process.

Various learning algorithms could be included in this process. Classical neural networks such as *K*-nearest neighbors algorithm [5] and generalized regression neural network [6] were adopted for their simplicity and generality. More sophisticated algorithms are needed for better predicting complicated data. Support vector machine (SVM) is one of the most popular nonlinear learning algorithms and has been widely used in feature selection [7–11]. Extreme learning machine (ELM) is one of the most popular single hidden layer feedforward networks (SLFN) [12]. It possesses faster calculation speed and better generalization ability than traditional artificial learning methods [13, 14], which highlights the advantages of employing ELM in feature selection, as reported in some studies [15–17].

In order to better locate optimal feature subsets, an efficient global search technique is needed. Particle swarm optimization (PSO) [18, 19] is an extremely simple yet fundamentally effective optimization algorithm and has produced encouraging results in feature selection [7, 20, 21]. Xue et al. considered feature selection as a multiobjective optimization problem [5] and firstly applied multiobjective PSO [22, 23] in feature selection. Some improved PSO such as hybridization of GA and PSO [9], micro-GA embedded PSO [24], and fractional-order Darwinian particle swarm optimization (FODPSO) [10] were introduced and achieved good performance in feature selection.

Training speed and optimization ability are two essential elements relating to feature selection. In this paper, we propose a novel feature selection method which employs ELM as learning algorithm and FODPSO as optimization algorithm. The proposed method is compared with SVM-based feature selection method in terms of training speed of learning algorithm and compared with traditional PSO-based feature selection method in terms of searching ability of optimization algorithm. And also, the proposed method is compared with a few well-known feature selection methods. All the comparisons are conducted on seven public regression datasets.

The remainder of the paper is organized as follows: Section 2 presents technical details about the proposed method.  Section 3 conducts the comparative experiments on seven datasets.  Section 4 makes conclusions of our work.

## 2. Proposed Method

### 2.1. Learning Algorithm: Extreme Learning Machine (ELM)

**Figure 1:**
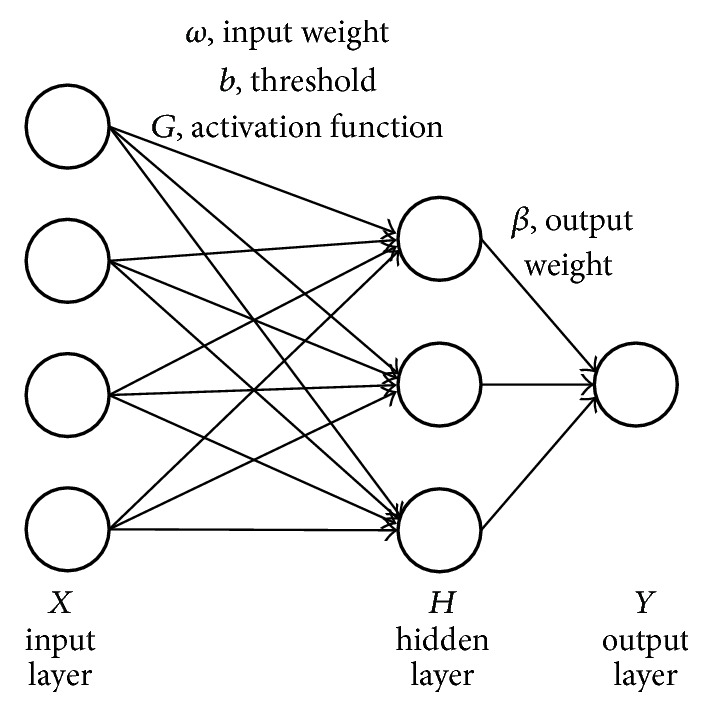
Schematic of extreme learning machine.

The schematic of ELM structure is depicted as Figure 1, where *ω* denotes the weight connecting the input layer and hidden layer and *β* denotes the weight connecting the hidden layer and output layer. *b* is the threshold of the hidden layer, and *G* is the nonlinear piecewise continuous activation function which could be sigmoid, RBF, Fourier, and so forth. *H* represents the hidden layer output matrix, *X* is the input layer, and *Y* is the expected output. Let $\bar{Y}$ be the real output; ELM network is used to choose appropriate parameters to make *Y* and $\bar{Y}$ as close to each other as possible.

(1) $$\begin{array}{c} min\left\|\;Y-\bar{Y}\;\right\|=min\left\|\;Y-H\beta \;\right\|. \end{array}$$

*H* is called the hidden layer output matrix, computed by *ω* and *b* as (2), in which $\tilde{N}$ denotes the number of hidden layer nodes and *N* denotes the dimension of input *X*:

(2) H=GωX+b=gω1·x1+b1⋯gωN~·x1+bN~⋮⋱⋮gω1·xN+b1⋯gωN~·xN~+bN~N×N~.

As rigorously proven in [13], for any randomly chosen *ω* and *b*, *H* can always be full-rank if activation function *G* is infinitely differentiable in any intervals. As a general rule, one needs to find the appropriate solutions of *ω*, *b*, *β* to train a regular network. However, given infinitely differentiable activation function, the continuous output can be approximately obtained through any randomly hidden layer neuron, if certain tuning hidden layer neuron could successfully estimate the output, as proven by universal approximation theory [24, 25]. Thus, in ELM, the only parameter that needs to be settled is *β*. *ω*, *b* can be generated randomly.

By minimizing the absolute numerical value in (1), ELM calculated the analytical solution as follows:

(3) $$\begin{array}{c} \hat{\beta }=H^{ƚ}Y, \end{array}$$

where *H*^*ƚ*^ is the Moore-Penrose pseudoinverse of matrix *H*. ELM network tends to reach not only the smallest training error, but also the smallest norm of weights, which indicates that ELM possesses good generalization ability.

### 2.2. Optimization Algorithm: Fractional-Order Darwinian Particle Swarm Optimization (FODPSO)

Kiranyaz et al. [19] developed a population-inspired metaheuristic algorithm named particle swarm optimization (PSO). PSO is an effective evolutionary algorithm which searches for the optimum using a population of individuals, where the population is called “swarm” and individuals are called “particles.” During the evolutionary process, each particle updates its moving direction according to the best position of itself (pbest) and the best position of the whole population (gbest), formulated as follows:

(4) Vit+1=ωVit+c1r1Pi−Xit+c2r2Pg−Xit,

(5) Xit+1=Xit+Vit+1,

where *X*_*i*_ = (*X*_*i*_^1^, *X*_*i*_^2^,…, *X*_*i*_^*D*^) is the particle position at generation *i* in the *D*-dimension searching space. *V*_*i*_ is the moving velocity. *P*_*i*_ denotes the cognition part called pbest, and *P*_*g*_ represents the social part called gbest [18]. *ω*, *c*, *r* denote the inertia weight, learning factors, and random numbers, respectively. The searching process terminates when the number of generation reaches the predefined value.

Darwinian particle swarm optimization (DPSO) simulates natural selection in a collection of many swarms [25]. Each swarm individually performs like an ordinary PSO. All the swarms run simultaneously in case of one trap in a local optimum. DPSO algorithm spawns particle or extends swarm life when the swarm gets better optimum; otherwise, it deletes particle or reduces swarm life. DPSO has been proven to be superior to original PSO in preventing premature convergence to local optimum [25].

Fractional-order particle swarm optimization (FOPSO) introduces fractional calculus to model particles' trajectory, which demonstrates a potential for controlling the convergence of algorithm [26]. Velocity function in (4) is rearranged with *ω* = 1, namely,

(6) Vit+1−Vit=c1r1Pi−Xit+c2r2Pg−Xit.

The left side of (6) can be seen as the discrete version of the derivative of velocity *D*^*α*^[*v*_*t*+1_] with order *α* = 1. The discrete time implementation of the Grünwald–Letnikov derivative is introduced and expressed as

(7) $$\begin{array}{c} D^{\alpha }\left[\;v_{t}\;\right]=\frac{1}{T^{\alpha }}\overset{r}{\underset{k=0}{\sum }}\frac{{\left(-1\right)}^{k}\Gamma \left(\alpha +1\right)v\left(t-kT\right)}{\Gamma \left(k+1\right)\Gamma \left(\alpha -k+1\right)}, \end{array}$$

where *T* is the sample period and *r* is the truncate order. Bring (7) into (6) with *r* = 4, yielding the following:

(8) Vit+1=αVit+α2Vit−1+α1−α6Vit−2+α1−α2−α24Vit−3+c1r1Pi−Xit+c2r2Pg−Xit.

Employ (8) to update each particle's velocity in DPSO, generating a new algorithm named fractional-order Darwinian particle swarm optimization (FODPSO) [27, 28]. Different values of *α* control the convergence speed of optimization process. The literature [27] illustrates that FODPSO outperforms FOPSO and DPSO in searching global optimum.

### 2.3. Procedure of ELM_FODPSO

**Figure 2:**
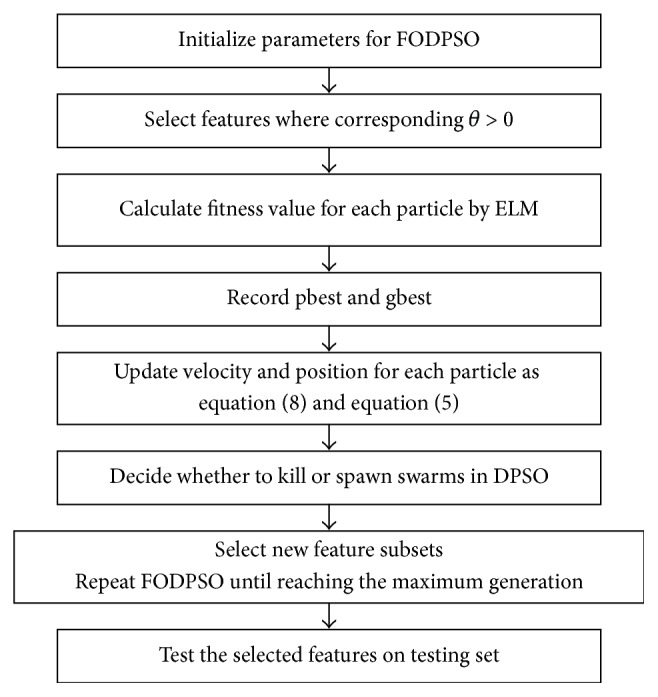
Procedure of the proposed methodology.

Each feature is assigned with a parameter *θ* within the interval [−1,1]. The *i*_th_ feature is selected when its corresponding *θ*_*i*_ is greater than 0; otherwise the feature is abandoned. Assuming the features are in *N*-dimensional space, *N* variables are involved in the FODPSO optimization process. The procedure of ELM_FODPSO is depicted in Figure 2.

## 3. Results and Discussions

### 3.1. Comparative Methods

Four methods, ELM_PSO [15], ELM_FS [29], SVM_FODPSO [10], and RReliefF [30], are used for comparison. All of the codes used in this study are implemented in MATLAB 8.1.0 (The MathWorks, Natick, MA, USA) on a desktop computer with a Pentium eight-core CPU (4 GHz) and 32 GB memory.

### 3.2. Datasets and Parameter Settings

Seven public datasets for regression problems are adopted, including four mentioned in [29] and additional three in [31], where ELM_FS is used as a comparative method. Information about seven datasets and the methods involved in comparisons are shown in Table 1. Only the datasets adopted in [29] can be tested by their feature selection paths; thus D5, D6, and D7 in Table 1 are tested by four methods except ELM_FS.

Each dataset is split into training set and testing set. 70% of the total instances are used as training sets if not particularly specified, and the rest are testing sets. During the training process, each particle has a series of feature coefficients *θ* ∈ [−1,1]. Hidden layer neurons number is set as 150, and kernel type as sigmoid. 10-fold cross-validation is performed to gain relatively stable MSE.

For FODPSO searching process, parameters are set as follows: *α* is formulated by (9), where *M* denotes the maximal iterations and *M* equals 200. Larger *α* increases the convergence speed in the early stage of iterations. Numbers of swarms and populations are set to 5 and 10, respectively. *c*_1_, *c*_2_ in (8) are both initialized by 2. We run FODPSO for 30 independent times to gain relatively stable results. Parameters for ELM_PSO, ELM_FS, SVM_FODPSO, and RReliefF are set based on former literatures.

(9) $$\begin{array}{c} \alpha =0.8-0.4\times \frac{t}{M},\;t=0,1,\ldots ,M. \end{array}$$

**Figure 3:**
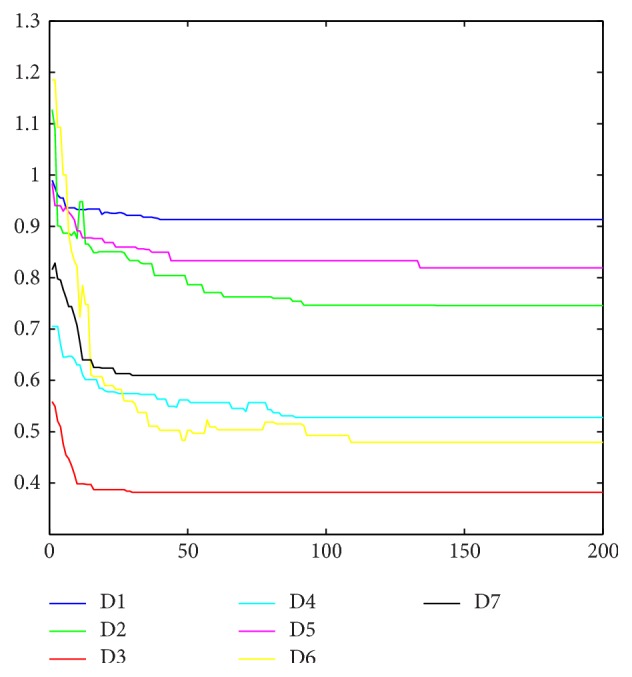
Convergence analysis of seven datasets.

Convergence rate is analyzed to ensure the algorithm convergence within 200 generations. The median of the fitness evolution of the best global particle is taken for convergence analysis, depicted in Figure 3. To observe convergence of seven datasets in one figure more clearly, the normalized fitness value is adopted in Figure 3, calculated as follows:

(10) $$\begin{array}{c} f_{Normolized}=\frac{{MSE}_{selected\_features}}{{MSE}_{all\_features}}. \end{array}$$

### 3.3. Comparative Experiments

**Figure 4:**
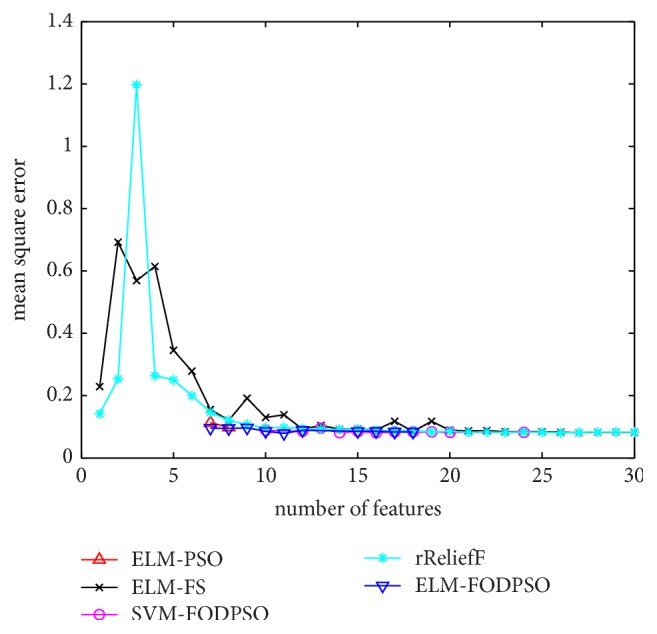
The evaluation results of Dataset 1.

**Figure 5:**
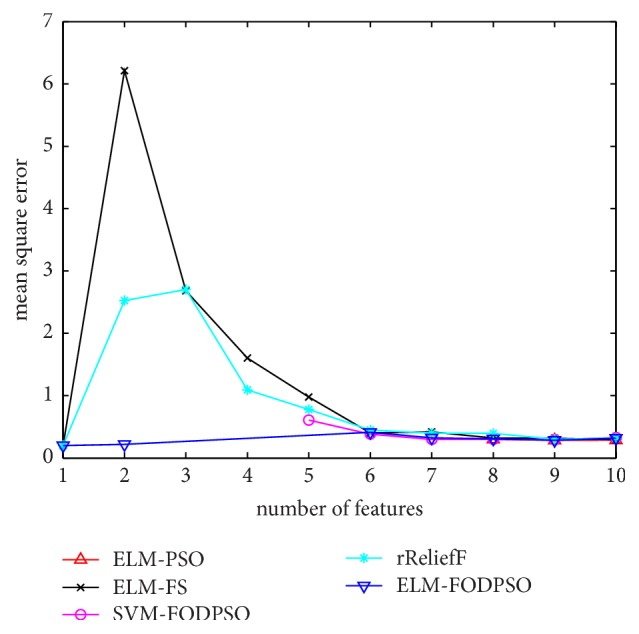
The evaluation results of Dataset 2.

**Figure 6:**
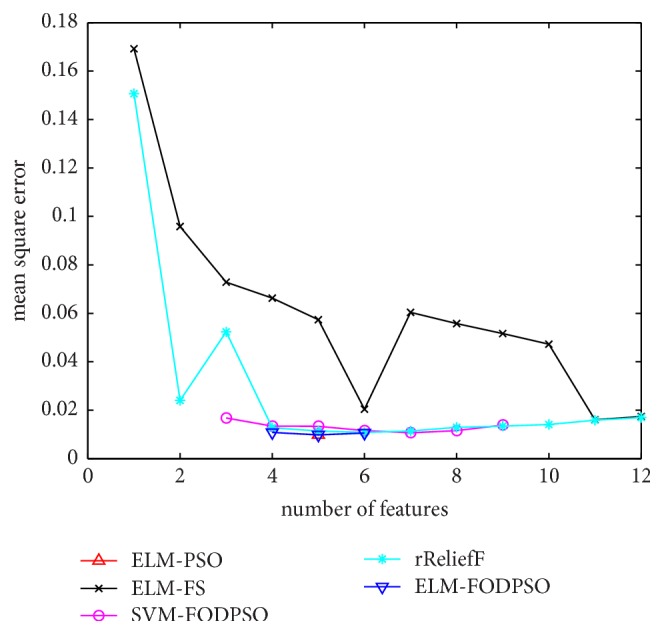
The evaluation results of Dataset 3.

**Figure 7:**
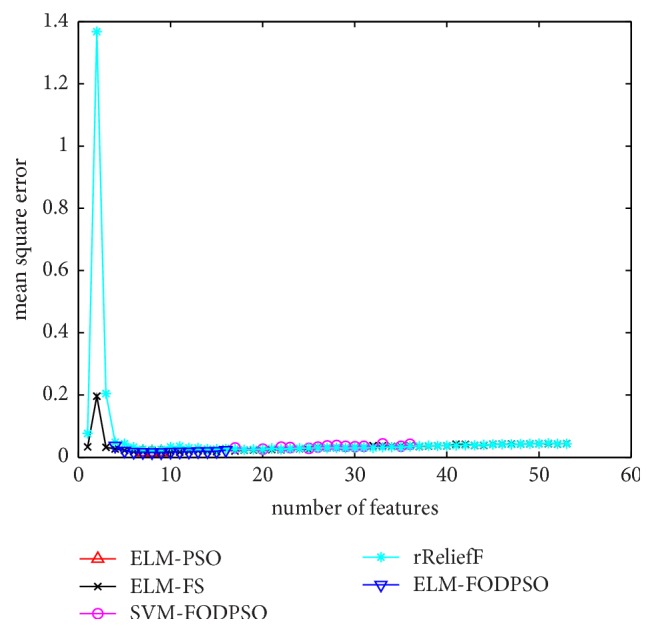
The evaluation results of Dataset 4.

**Figure 8:**
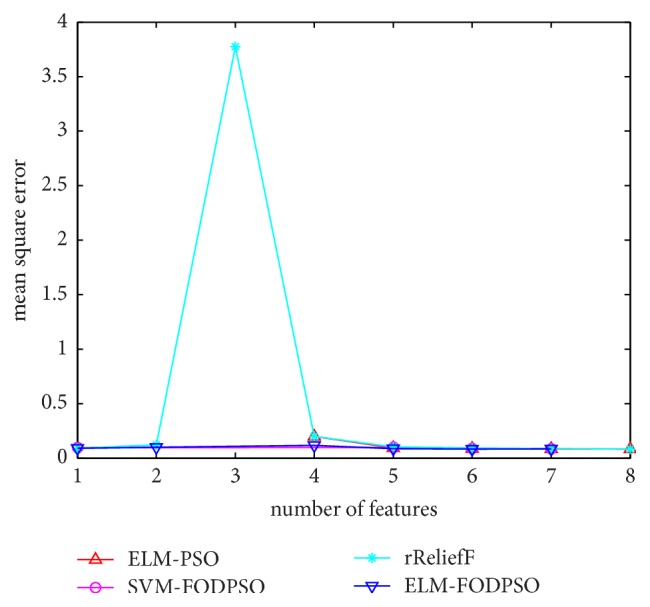
The evaluation results of Dataset 5.

**Figure 9:**
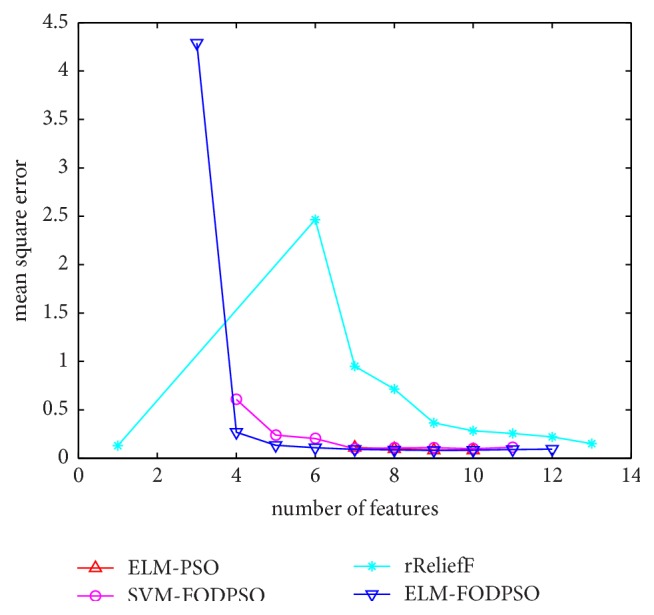
The evaluation results of Dataset 6.

**Figure 10:**
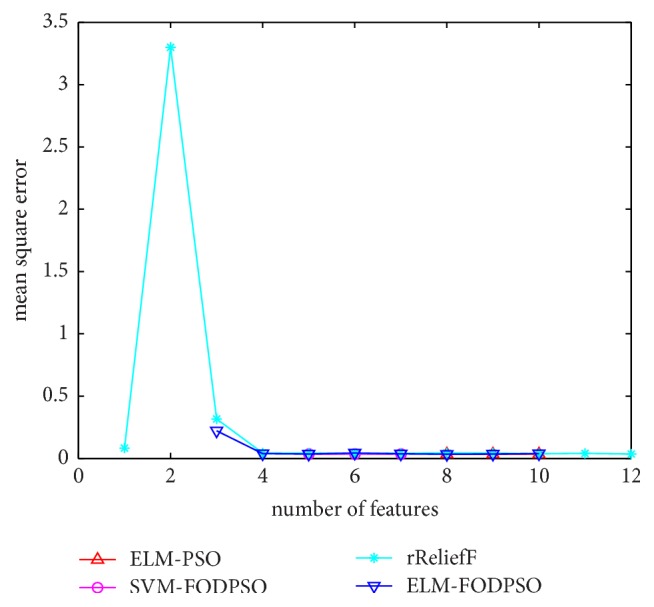
The evaluation results of Dataset 7.

In the testing set, MSE acquired by ELM is utilized to evaluate performances of four methods. For all the methods, the minimal MSE is recorded if more than one feature subset exists in the same feature scale. MSEs of D1–D7 are depicted in Figures 4–10, respectively. The *x*-axis represents increasing number of selected features, while the *y*-axis represents the minimum MSE value calculated with features selected by different methods at each scale. Feature selection aims at selecting smaller feature subsets to obtain similar or lower MSE. Thus, in Figures 4–10, the closer one curve gets to the left corner of coordinate, the better one method performs.

ELM_FODPSO and SVM_FODPSO adopt the same optimization algorithm, yet employ ELM and SVM as learning algorithm, respectively. For each dataset, training time of ELM and SVM is obtained by randomly running them 30 times in two methods; the averaged training time of ELM and SVM in seven datasets is recorded in Table 2. It is observed that ELM acquires faster training speed in six of seven datasets. Compared with SVM, single hidden layer and analytical approach make ELM more efficient. Faster speed of ELM highlights its use in feature selection due to many iterative actions involved in FODPSO.

ELM_FODPSO, ELM_PSO, and ELM_FS adopt the same learning algorithm, yet employ FODPSO, PSO and Gradient Descent Search as optimization algorithms, respectively. For D1, D2, and D3, ELM_FODPSO and ELM_PSO perform better than ELM_FS; the former two acquire lower MSE than ELM_FS under similar feature scales. For D4, three methods get comparable performance.

Table 3 shows the minimum MSE values acquired by five methods and the corresponding numbers of selected features, separated by a vertical bar. The last column represents the MSE values calculated by all features and the total number of features. The lowest MSE values on each dataset are labeled as bold. Among all datasets, ELM_FODPSO obtains six lowest MSE values, ELM_PSO obtains two, and RReliefF obtains one. For D3, ELM_FODPSO and ELM_PSO get comparable MSE values by the same feature subset; therefore, 0.0099 and 0.0098 are both labeled as lowest MSE values. For D5, ELM_PSO and RReliefF get the lowest MSE 0.0838 using all the 8 features and ELM_FODPSO gets a similar MSE 0.0841 with only 6 features.

## 4. Conclusions

Feature selection techniques have been widely studied and commonly used in machine learning. The proposed method contains two steps: constructing fitness functions by ELM and seeking the optimal solutions of fitness functions by FODPSO. ELM is a simple yet effective single hidden layer neural network which is suitable for feature selection due to its gratifying computational efficiency. FODPSO is an intelligent optimization algorithm which owns good global search ability.

The proposed method is evaluated on seven regression datasets, and it achieves better performance than other comparative methods on six datasets. We may concentrate on exploring ELM_FODPSO in various situations of regression and classification applications in the future.

## Figures and Tables

**Table 1:** Information about datasets and comparative methods. A1, A2, A3, A4, and A5 represent ELM_PSO, ELM_FS, SVM_FODPSO, RReliefF, and ELM_FODPSO, respectively.

Label	Dataset	Number of instances	Number of features	Comparative methods
D1	Poland	1370	30	A1, A2, A3, A4, A5
D2	Diabetes	442	10	A1, A2, A3, A4, A5
D3	Santa Fe Laser	10081	12	A1, A2, A3, A4, A5
D4	Anthrokids	1019	53	A1, A2, A3, A4, A5
D5	Housing	4177	8	A1, A3, A4, A5
D6	Abalone	506	13	A1, A3, A4, A5
D7	Cpusmall	8192	12	A1, A3, A4, A5

**Table 2:** Running time of SVM and ELM on seven datasets.

Running time (s)	D1	D2	D3	D4	D5	D6	D7
SVM	0.021	**0.002**	0.612	0.016	0.093	0.045	0.245
ELM	**0.018**	0.009	**0.056**	**0.013**	**0.027**	**0.010**	**0.051**

**Table 3:** Minimum MSE values and the corresponding number of selected features.

Dataset	Method					
	ELM_PSO	ELM_FS	SVM_FODPSO	RReliefF	ELM_FODPSO	all features
MSE N. feature						
D1	0.0983∣8	0.0806∣27	0.0804∣14	0.0804∣26	**0.0791**∣**11**	0.0820∣30
D2	0.2844∣9	0.2003∣1	0.2919∣9	0.2003∣1	**0.1982∣1**	0.3172∣10
D3	**0.0099∣5**	0.0160∣11	0.0106∣7	0.0108∣6	**0.0098∣5**	0.0171∣12
D4	0.0157∣8	0.0157∣9	0.0253∣20	0.0238∣18	**0.0156∣7**	0.0437∣53
D5	**0.0838∣8**	—	0.0853∣7	**0.0838∣8**	0.0841∣6	**0.0838∣8**
D6	0.0827∣10	—	0.0981∣7	0.1292∣1	**0.0819∣9**	0.1502∣13
D7	0.0339∣9	—	0.0343∣6	0.0355∣12	**0.0336∣8**	0.0355∣12
